# Can improving law enforcement effectively curb illegal land use in China?

**DOI:** 10.1371/journal.pone.0246347

**Published:** 2021-02-11

**Authors:** Siyi Chen, Zhigang Chen, Yan Shen

**Affiliations:** 1 School of Geography and Ocean Science, Nanjing University, Nanjing, China; 2 Key Laboratory of Coastal Zone Exploitation and Protection, Ministry of Natural Resources of China, Nanjing, China; Institute for Advanced Sustainability Studies, GERMANY

## Abstract

In view of the current severe situation of illegal land use in China, it is of great significance to explore the impact of the evolution and promotion of land law enforcement effectiveness, which will improve China’s land law enforcement system and effectively curb illegal land use. This paper explains the changes and enhancements of the effectiveness of land law enforcement since the implementation of China’s National Land Supervision System in terms of the deterrence, difficulty, and strength of land law enforcement, and explores the role of land law enforcement effectiveness in changing illegal land use behaviors from a theoretical level. Then, a corresponding empirical test was carried out using the provincial panel data of Mainland China from 2007 to 2016. The results show that the increase in land law enforcement deterrence and strength will help reduce the number of illegal land use cases, but it will drive the lawbreakers to "commit major cases in desperation", leading to the increase of the degree of illegal land use; and with the decrease of land law enforcement difficulty, the degree of illegal land use will be significantly reduced. At the end of this paper, several policy suggestions are put forward to effectively curb the illegal land use from the perspective of improving the land law enforcement system and enhancing the effectiveness of law enforcement.

## 1. Introduction

Since the reform and opening up, China’s economic and social development has made remarkable achievements. To a certain extent, this can be attributed to the fact that the Chinese government has manifested the land value through institutional innovation [1–3], and provided land (space) for the rapid development of industrialization and urbanization. In China, the important role of land in supporting economic and social development and accumulating wealth has long been widely known [4–8]. There has even been an economic competition to sell land [9–11]. However, due to the large population, the Chinese government has implemented strict target control on the scale of land used for non-agricultural construction based on the goal of ensuring food security [12, 13]. Facing the temptation of huge economic interests, it is difficult to prevent illegal land activities represented by illegal occupation of land for non-agricultural construction [10, 14–16]. The Land Management Law of the People’s Republic of China (hereinafter referred to as the "Land Management Law") has been implemented since 1987, although it has clear regulations on the investigation and punishment of illegal land use, it has had little effect. According to statistics, from 1999 to 2005, the area of illegal land use cases reached about 28,000 hectares per year, and the number of illegal cases basically remained at the level of 100,000 per year [17]. This prompted the Chinese government to further strict land law enforcement, formulate and implement a series of policies, use advanced technologies such as satellite remote sensing monitoring, and in 2006 proposed the "National Land Supervision System" (hereinafter referred to as the NLSS, formally implemented in 2007). It can be said that since the late 1980s, the Chinese government has made a lot of efforts to improve the land law enforcement system and enhance the effectiveness of land law enforcement. But how exactly do these efforts pay off? Has the increased effectiveness of land law enforcement produced the expected suppressive effect on illegal land use? It still needs to be thoroughly analyzed and judged.

In recent years, scholars have been paying close attention to revealing the reasons behind illegal land use in China and exploring effective ways to curb illegal land use. Many existing studies have pointed out that since land development is closely related to the interests of local governments [18–22], illegal activities are mainly driven by local land finance and regional economic development [23–25]. Therefore, breaking the dependence of local governments on land economy will help to curb illegal land use. In addition, the improvement of technical means and law enforcement system are also considered as two important ways to curb land violations. On the one hand, the development of the land law enforcement monitoring system supported by UAV remote sensing technology and geographic information technology, the mobile supervision system of land and resources and other technical means can more effectively monitor and identify illegal land use behaviors [26–28]. On the other hand, measures such as reforming law enforcement ideas, clarifying punishment provisions, and strengthening the enforcement of land regulations can better maintain the order of land management and combat land violations [29]. Among them, the NLSS officially implemented in 2007 is undoubtedly an important part of the improvement of the land law enforcement system. In this regard, many scholars also specifically discussed the effects of the implementation of the NLSS. Relevant research finds that the implementation of the NLSS (including routine land supervision and special land supervision) promotes the land law enforcement, has a significant suppressive effect on the number of illegal land cases and areas involved [30, 31], and has a certain degree of cultivated land protection effect [32]. Of course, the existing literature mainly takes the impact of the implementation of the NLSS as a whole (or a single policy event) in terms of the effectiveness of land law enforcement. In fact, with the introduction and implementation of the NLSS, China’s land law enforcement effectiveness is improved in many aspects. Therefore, to explore the improved effectiveness of land law enforcement on curbing illegal land use behavior also needs to be based on a more comprehensive perspective.

This paper will focus on the change of land law enforcement effectiveness in China since the implementation of the NLSS in 2007, and systematically explain the specific impact of improving land law enforcement effectiveness on curbing illegal land use from three aspects: land law enforcement deterrence, land law enforcement difficulty, and land law enforcement strength. The remaining parts of this paper are arranged as follows: Section two will discuss the theoretical impact of the improvement of land law enforcement effectiveness on illegal land use based on sorting out the evolution of China’s land law enforcement effectiveness, and propose corresponding research hypotheses. In section three, based on theoretical analysis, an econometric model that reflects the impact of changes in land law enforcement effectiveness on illegal land use will be constructed, and the relevant statistical data will be used to initially explore the relationship between the improvement of land law enforcement effectiveness and changes in illegal land use. In section four, panel data of 31 provincial regions in Mainland China from 2007 to 2016 will be used for model estimation and empirical testing, and in-depth analysis of the results will be given. In the last section, research conclusions and policy implications will be provided.

## 2. Theoretical analysis and research hypothesis

### 2.1 The institutional evolution and effectiveness improvement of land law enforcement in China

The official law enforcement of the Chinese government in the field of land use stems from the growing emphasis on land management in the mid-1980s and the formal enactment of the Land Management Law. Since then, with the change of the land use situation, especially the spread of illegal land use, the relevant law enforcement system and policies have been continuously enriched and improved, and the practice of land law enforcement has been gradually standardized and normalized. In summary, the development of China’s land law enforcement system and corresponding practice has mainly experienced the following three stages. (1) The first stage is from the mid-1980s to the mid-1990s: China’s land law enforcement system has grown from scratch, and law enforcement practice has begun to get on track. This stage began with the issuance of the No. 7 document of the Central Committee of the Communist Party of China in 1986, "*Notice on Strengthening Land Management and Stopping Arbitrary Occupation of Cultivated Land*." The notice called for the rapid formulation of the "Land Management Law of the People’s Republic of China" and the decision to establish the National Bureau of Land Administration. Subsequently, the Land Management Law was passed and officially implemented the following year. In the same period, the National Bureau of Land Administration was established, marking that the land-use behavior has laws to follow and violations will be investigated. Since then, the National Bureau of Land Administration has also promulgated the Interim Provisions for Land Supervision in 1995, which has further refined the responsibility for supervision and inspection. (2) The second stage is from the late 1990s to the beginning of the 21^st^ century: in response to new problems arising from practice, the land law enforcement system checks for and fills in gaps, and the law enforcement practice is further refined and standardized. In the late 1980s, although land law enforcement started to work, due to the rapid urbanization of China at that time [33], the sharp increase in the value of land assets led to a large amount of indiscriminate occupations of cultivated land and illegal land expropriations [34]. In this regard, the No. 11 document of the Central Committee of the Communist Party of China in 1997, "*Circular of the Central Committee of the Communist Party of China and the State Council on Further Strengthening Land Management and Effective Protection of Cultivated Land*", proposed that all kinds of non-agricultural construction land should be inventoried and rectified. It also emphasized the need to establish and improve the land law enforcement supervision system and strengthen the supervision practice. In 1998, the Land Management Law was revised accordingly, and the Land Management Law Implementation Regulations were also issued in the same year. From 1999 to 2001, the Ministry of Land and Resources successively issued a series of documents, including the "*Notice on Issues Related to the Implementation of the Dynamic Supervision Responsibility System in Land Law Enforcement and Supervision Work*", "*Notice on the Use of Satellite Remote Sensing Monitoring Technology to Carry out Land Law Enforcement Supervision Work*" and "*Notice on Printing and Distributing the Three Systems including the Interrogation System for Cases of Illegal Land Use*", to standardize land law enforcement. (3) The third stage is from the beginning of the 21^st^ century to the present: the NLSS has been formally implemented, and the land law enforcement system has been improved. At this stage, in the context of China’s "Development Zone Fever" continuing to heat up, the State Council issued the "*Emergency Notice on Strengthening Work to Further Govern and Rectify the Order of the Land Market*" in 2003, which calls for resolutely implementing the cleaning up and rectification of development zones and seriously investigating and punishing land violations. Subsequently, based on summarizing the practice of clearing the development zone and rectifying the land market, the State Council issued Document No. 28 in 2004 on “*Decisions on Deepening Reform and Strict Land Management*”, which called for strengthening supervision of land law enforcement and establishing the NLSS. In 2005, the Ministry of Land and Resources issued the “Standards for Investigating and Handling Land Illegal Acts”, which aims to strengthen law enforcement. In 2006, the General Office of the State Council issued Circular No. 50 "*Notice on Issues Related to the Establishment of the NLSS*". The NLSS was formally introduced and began to be implemented the following year. Since then, land law enforcement has tended to be standardized and normalized.

It should be said that with the implementation of the NLSS and the improvement of the law enforcement system, the effectiveness of land law enforcement in China has been significantly improved. According to statistics, in 2007, based on the opinions and suggestions put forward by the land supervision agencies, the relevant local governments in China revoked 63 illegal development parks and their management committees, returned more than 166.67 hectares of illegally occupied land, processed more than 15333.33 hectares of idle land, and 256 relevant responsible persons were transferred to the judicial organ for criminal responsibility [35]. In recent years, the effectiveness of land law enforcement in China has been improved in many aspects: first, the deterrence of land law enforcement has been enhanced. Deterrence refers to people’s fear of potential punishment [36]. In general, the closer the distance between law enforcers and potential violators, the stronger the law enforcement deterrence. In China, the early land management system is characterized by "combination of blocks and blocks", that is, the land approval authority and the personnel rights of leading cadres are mainly managed by local governments and committees of CPC (the Communist Party of China), and local administrative department of land and resources are subordinate departments of local governments [37]. Under this system, the land law enforcement deterrence of the relevant departments is actually very low. When the local government decides to implement or condone illegal land use, it is difficult for these subordinate departments to exert substantial restraint on it. In response to this phenomenon, the State Council of China issued the “*Notice on Issues Regarding the Reform of the Land and Resources Management System Below the Provincial Level*” in 2004, marking that China began to implement the reform of land vertical management system (local administrative department should accept the leadership of the corresponding higher-level department), and the law enforcement deterrence of relevant departments has been strengthened to a certain extent. However, this reform only vertically manages institutions below the provincial level, and there is still a lack of deterrence in land law enforcement between the central and provincial governments. With the implementation of the NLSS, the central government has set up nine National Land Supervision Bureaus stationed in local areas, covering the whole mainland China (Table 1) [38]. According to the system design, these bureaus supervise and inspect the land use and management behaviors of local governments on behalf of the central government. Through these local posts directly under the central government, the central government has realized the change from "far away in the sky" to "near at hand" in the supervision of local land violations, and effectively strengthened the law enforcement deterrence on local potential land violators. Second, the difficulty of land law enforcement is reduced. The difficulty of land law enforcement is mainly reflected in time and space. In general, under certain circumstances of law enforcement personnel, the higher the frequency of violations, the greater the difficulty of law enforcement; the larger the regulatory area, the greater the difficulty of law enforcement. The land law enforcement work in China has been carried out for many years. Due to the continuous improvement and refinement of relevant laws and regulations and the technical improvement of illegal land use monitoring, the difficulty of land law enforcement has been greatly reduced. For example, in recent years, the development of UAV remote sensing technology, land law enforcement monitoring system supported by geographic information technology, land and resources mobile inspection system, etc. has made monitoring and identification of illegal land use more effective [26–28]; In addition, the implementation of the NLSS has also made a great contribution to the reduction of the difficulty of land law enforcement. Since each National Land Supervision Bureau stationed in the local area is in charge of three to five provinces (or municipalities directly under the central government), compared with the "one to many" management mechanism of the central government for each local before the implementation of the NLSS, the "many to many" land management mechanism after the implementation of the system effectively promotes the subdivision of land supervision scope, thus reducing the difficulty of regional land law enforcement. Third, the strength of land law enforcement has been increased. The strength of land law enforcement is reflected in whether the land law enforcement department implements the "law violations must be investigated" work guidelines when discovering illegal land use. Under the circumstances that the punishment measures (i.e., the severity of the punishment) for relevant violations are determined by law, increasing the probability of punishment means enhancing the law enforcement [39]. It should be said that in recent years, especially with the implementation of the NLSS, the rate of registered land violation cases across China has increased significantly. This also fully demonstrates that China’s current land law enforcement strength has increased significantly.

**Table 1:** National Land Supervision Bureaus and their supervision scopes.

National Land Supervision Bureau	Scope of Supervision
	(Provincial or Municipal Area)
Beijing Bureau of National Land Supervision	Beijing, Tianjin, Hebei, Shanxi, and Inner Mongolia
Shenyang Bureau of National Land Supervision	Liaoning, Jilin, Heilongjiang, and Dalian
Shanghai Bureau of National Land Supervision	Shanghai, Zhejiang, Fujian, Ningbo, and Xiamen
Nanjing Bureau of National Land Supervision	Jiangsu, Anhui, and Jiangxi
Jinan Bureau of National Land Supervision	Shandong, Henan, and Qingdao
Guangzhou Bureau of National Land Supervision	Guangdong, Guangxi, Hainan, and Shenzhen
Wuhan Bureau of National Land Supervision	Hubei, Hunan, and Guizhou
Chengdu Bureau of National Land Supervision	Chongqing, Sichuan, Yunnan, and Tibet
Xi’an Bureau of National Land Supervision	Shaanxi, Gansu, Qinghai, Ningxia, Xinjiang and Xinjiang Production and Construction Corps

### 2.2 The impact of the improvement of land law enforcement effectiveness on illegal land use

Just like ordinary criminal acts, from the perspective of economics, illegal land use can also be regarded as an economic act based on the "cost-benefit" decision [40]. Lawbreakers gain certain illegal benefits and social values through their illegal actions, which damage the interests of other members of society; of course, they also need to pay certain costs, including time, early preparation, the possible benefits of giving up legal activities, and legal sanctions they may suffer after breaking the law. Currently in China, the main bodies of illegal land use include local governments, village collectives, enterprises, and individuals. The pursuit of land interests by relevant organizations or individuals, the fiscal revenue demand, and the competition for economic growth of local governments [23, 41–44] have been regarded as the main factors driving illegal land use. Through the implementation of illegal acts, those organizations or individuals can obtain huge economic and social benefits.

The decision-making of the illegal activities of the relevant parties depends on how they define the relationship between illegal land benefit and its cost. Once they believe that the expected benefit of illegal land use is higher than the cost they need to pay, then as rational economic person, they will decide to break the law. From the perspective of local governments, the cost of breaking the law mainly includes the compensation for farmers whose interests are damaged, the adverse impact on the local long-term development, and the possible punishment and loss of political future of relevant public officials. For those village collectives, enterprises or individuals, the cost of illegality mainly includes the time (or labor) and capital input for illegal land use, and the possible fines and even criminal punishment after investigation. The changes in land law enforcement actions and effectiveness will have an important impact on their cost of breaking the law. Because the fixed part of cost (such as economic compensation, time or labor, capital investment, etc.) that the lawbreakers need to pay for illegal land use will not be higher than that of legal use of land; and once the effectiveness of land law enforcement increases, the possibility of being investigated and punished increases, then the uncertain cost due to legal punishment will be very high, which often exceeds the cost of their legal land use, and may even make the cost outweigh the benefit.

**Fig 1:**
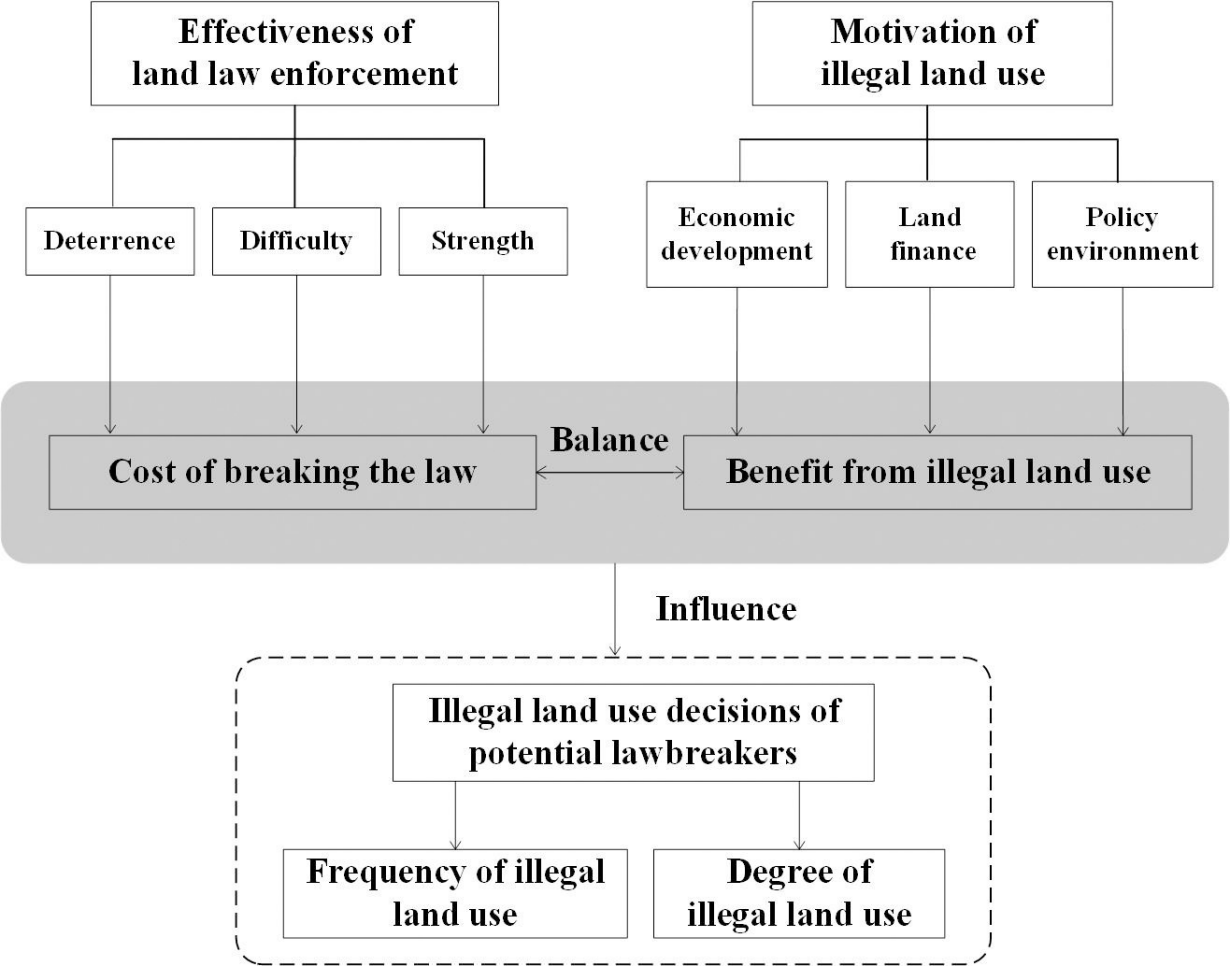
The mechanism of the impact of land law enforcement effectiveness on illegal land use.

As mentioned above, China’s land law enforcement system and practice have been continuously improved since the mid-1980s. Especially after the implementation of the NLSS, the effectiveness of land law enforcement has been significantly improved: the deterrence of land law enforcement has been enhanced, the difficulty of land law enforcement has been reduced, and the strength of land law enforcement has been increased. For the main bodies who may break the law, this means a big increase on the probability of being investigated and punished. The resulting increase in the cost of illegality will inevitably prompt the relevant parties to weigh the gains and losses and make corresponding decisions. Therefore, under the condition that the illegal gains are basically unchanged, the relevant lawbreakers, including local governments, village collectives, enterprises and individuals, will try to reduce the cost of illegality as much as possible, so that the expected benefit is greater than the cost before the illegal acts are implemented. Because the cost of illegal land use is positively correlated with the frequency of violations and the risk of being investigated and dealt with; that is to say, the higher the frequency of illegal acts, the higher the risk of single violation being investigated, the higher the cost of land violations. Therefore, the lawbreakers tend to balance their illegal costs and benefits by reducing the frequency of illegal activities and increasing the scale of land use involved in a single violation (i.e. increasing the degree of illegal land use and committing major cases). In addition, in some cases, if those lawbreakers find it impossible to reduce the cost of illegal activities by certain means, they may choose to give up land violations, thus reducing the occurrence of cases (Fig 1). Based on the above analysis, combined with the specific performance of the improvement of land law enforcement effectiveness, we can condense the impact of the improvement of land law enforcement effectiveness on illegal land use into the following four hypotheses:

1. From the perspective of the impact of enhanced land law enforcement deterrence on illegal land use, since the central government has selected only nine provinces (or municipalities) in the country to establish National Land Supervision Bureaus, which are respectively located in Beijing, Liaoning, Shanghai, Jiangsu, Shandong, Guangdong, Hubei, Sichuan and Shaanxi provinces, there are actually two major regions in the country where the National Land Supervision Bureaus reside or not. In theory, it may occur that the former is closer to the central government agency, has more access to relevant information, then has stronger land law enforcement deterrence [45]. Therefore, it can be considered that when other factors affecting the degree of land law enforcement deterrence are relatively consistent, the closer the area to the central government agency, the stronger the land law enforcement deterrence and the fewer cases of illegal land use (Hypothesis 1).
2. From the perspective of the impact of reduced difficulty in land law enforcement on illegal land use, due to land supervision and law enforcement requires a certain amount of labor power, material, and financial input, under the constraint of relevant inputs, when the actual area of the supervision is larger, the corresponding difficulty of land law enforcement will be greater. Therefore, although the establishment of multiple local bureaus has reduced the overall land law enforcement difficulty by subdividing the scope of supervision, the administrative land area difference of different provinces or regions will lead to the difference of land law enforcement difficulty, thus affecting the effectiveness of land law enforcement. So, it can be considered that when other factors affecting the difficulty of land law enforcement are relatively consistent, the larger the jurisdiction area, the greater the difficulty of land law enforcement and the greater the number of illegal land use cases (Hypothesis 2).
3. From the perspective of the impact of increased land law enforcement strength on illegal land use, increasing the registered rate of illegal cases can reduce or even eliminate the fluke mentality that potential violators may have on escaping punishment after breaking the law, and form a general awareness of the law-abiding in the whole society, so as achieve the purpose of reducing illegal activities. Therefore, it can be considered that under the circumstances that other factors affecting the strength of land law enforcement are relatively consistent, the higher the annual registered rate of illegal cases is, the greater the strength of land law enforcement and the lower the number of illegal land use cases (Hypothesis 3).
4. Increased deterrence, reduced difficulty and increased strength in land law enforcement will undoubtedly greatly increase the risk of illegal land use entities. In consideration of hedging the increased risk of illegality, the relevant organizations or individuals are likely to reduce the frequency of illegality and increase the scale of land use involved in a single violation, in order to balance the expected net income of their illegal land use. Therefore, it can be considered that as the effectiveness of land law enforcement increases, that is, the enhancement of deterrence, the reduction of difficulty and the increase of strength, the degree of illegal land use will increase (Hypothesis 4).

## 3. Model and data

### 3.1. Model

Based on the above theoretical analysis, this paper constructs the econometric model shown in Eq (1) to test the specific impact of land law enforcement effectiveness change (mainly the improvement of land law enforcement effectiveness since the implementation of the NLSS) on the number of case and the degree of illegal land use. In addition, given that many relevant researches have pointed out that other factors, such as local economic development, population change, and land marketization, have significant impacts on illegal land use [41, 46, 47], these factors are also added in the model as control variables.

NUMijDEGij=c+a1*BURij+a2*ADAij+a3*RRCij+a4*GPIij+a5*TPOij+a6*MARij+ε (1)

Wherein, *NUM*_*ij*_ and *DEG*_*ij*_ are the dependent variables reflecting the number of illegal land cases (measured by the number of illegal land cases occurred in this year) and the degree of illegal land use (measured by the average area involved in a single illegal land use case) respectively. Among the explanatory variables, *BUR*_*ij*_ is a dummy variable reflecting land law enforcement deterrence in area i during period j, based on whether the area is the stationed region of National Land Supervision Bureau (the value of the stationed region is 1, and the value of the other region is 0); *ADA*_*ij*_ is a variable that reflects the difficulty of land law enforcement in area i during period j, measured by the regional administrative land area; *RRC*_*ij*_ is a variable that reflects the strength of land law enforcement in area i during period j, measured by the proportion of the number of cases registered in this year (i.e. the registered rate). Among the control variables, *GPI*_*ij*_ is a variable that reflects the economic development in area i during period j, measured by the regional GDP index; *TPO*_*ij*_ is a variable that reflects the population situation in area i during period j, measured by the total population at the end of the year. *MAR*_*ij*_ is a variable that reflects the degree of land marketization in area i during period j, measured by the proportion of the land area for public tender, auction and listing of quotation in the total land area for sold. ɑ_1_ to ɑ_6_ are the coefficients of the corresponding variables in the model; c and ε are the constant term and the random disturbance term of the model respectively.

### 3.2 Data

The panel data from 31 provinces (or autonomous regions, municipalities) in Mainland China from 2007 to 2016 are used to estimate the above model. The specific data sources are as follows: the data of variable NUM is directly from the China Land and Resources Yearbook (2008–2017) [48]; the data of variable DEG is calculated by the number of cases and corresponding illegal area occurred in one year provided in China Land and Resources Yearbook (2008–2017), and is measured by the average area involved in each case [49]; The data of variable RRC is obtained by calculating the number of cases occurred and the number of cases registered in one year provided in China Land and Resources Yearbook (2008–2017); The data of variable MAR is obtained through the calculation of the sold area of state-owned land and the area for public tender, auction and listing of quotation provided in China Land and Resources Yearbook (2008–2017); The data of variables GPI and TPO are directly from China Statistical Yearbook (2008–2017) [50]; the data of variable BUR refers to the relevant documents of the NLSS and is obtained by direct assignment; the data of variable ADA, which refers to land area, is mainly from the provincial statistical yearbooks or relevant government websites (collected in Baidu Baike and available online. https://baike.baidu.com/item/%E4%B8%AD%E5%9B%BD%E5%90%84%E7%9C%81%E6%A6%82%E5%86%B5/15285626?fr=aladdin). Table 2 shows the descriptive statistics of relevant variables in the above model.

**Table 2:** Descriptive statistics of each variable listed in the model.

**Variable**	**Unit**	**Number of observations**	**Mean**	**Std. deviation**	**Minimum**	**Maximum**
NUM	case	310	1832.58	2214.00	0	12485
DEG	ha/case	310	0.73	0.78	0	8.39
BUR	—	310	0.29	0.45	0	1
ADA	10,000	310	31.05	38.20	0.63	166.49
	km^2^					
RRC	—	310	0.63	0.30	0	1.11
GPI	—	310	110.91	2.90	97.5	119.1
TPO	10,000 people	310	4333.29	2734.36	288.83	10999
MAR	—	310	0.83	0.17	0.09	0.99

### 3.3 Relationship between the improvement of land law enforcement effectiveness and changes in illegal land use

**Fig 2:**
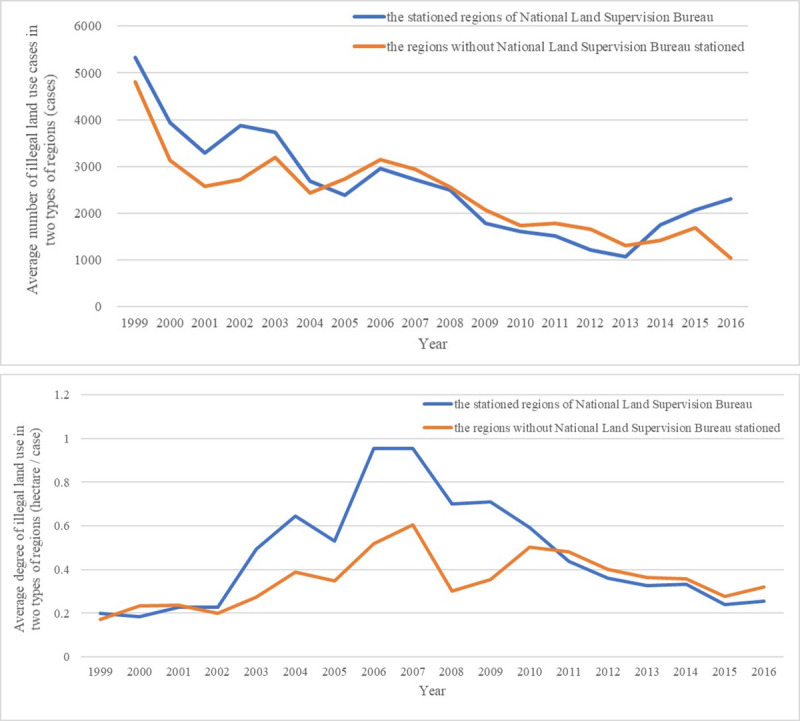
Land law enforcement deterrence and illegal land use.

First of all, it is not difficult to find that the number of cases and the degree of illegal land use in the two types of regions after the implementation of the NLSS (after 2007) have a certain downward trend (Fig 2), which shows that the increased deterrence of land law enforcement does have a certain curbing effect on illegal land use.

**Fig 3:**
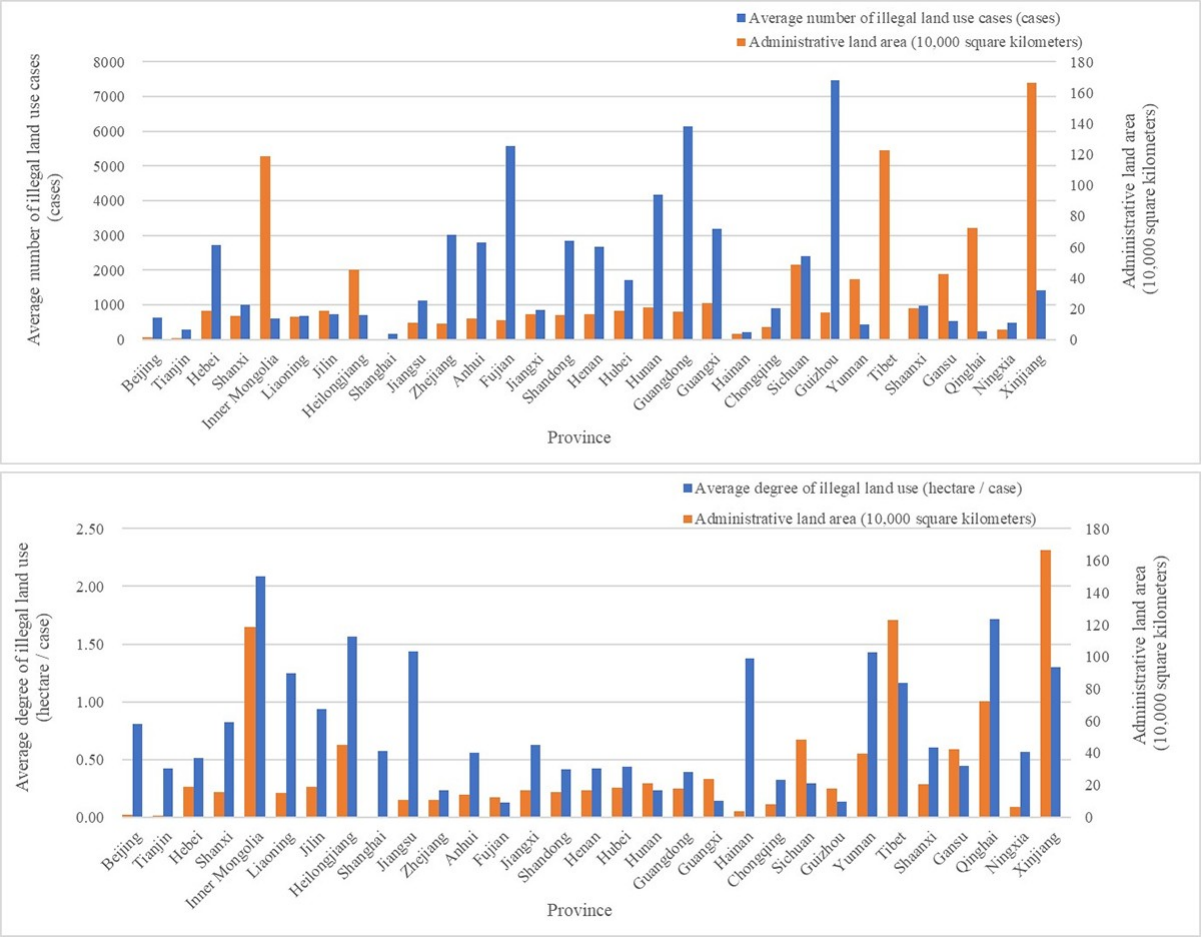
Land law enforcement difficulty and illegal land use.

Secondly, while characterizing the difficulty of land law enforcement by regional administrative land area, after analyzing the changes of the number of cases and the degree of illegal land use after the implementation of the NLSS, it can be found that the relationship between the difficulty of land law enforcement and the number of illegal land use cases is not significant, but there is a relatively obvious consistent change trend between the difficulty of land law enforcement and the degree of illegal land use (Fig 3).

**Fig 4:**
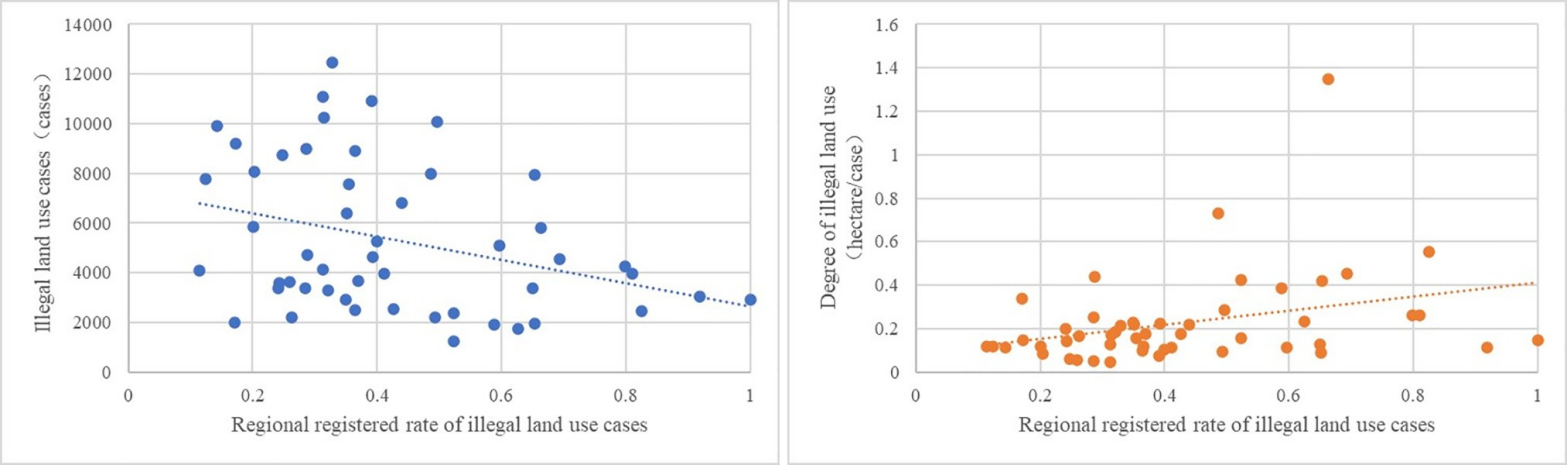
Land law enforcement strength and illegal land use.

Thirdly, from the perspective of the relationship between land law enforcement strength and changes in illegal land use, by analyzing the data from the five regions with the most serious illegal land use situation since the implementation of the NLSS-Guizhou Province, Guangdong Province, Fujian Province, Hunan Province, and Guangxi Province (Top 5 in cumulative number of illegal land use cases from 2007 to 2016), we can find that there is a certain correlation between the registered rate and the number of cases and the degree of illegal land use(Fig 4). Of course, further model test is needed to clarify the impact of changes in land law enforcement strength on illegal land use.

## 4. Results

We used STATA14.0 software to estimate the above econometric model, using the number of cases and the degree of illegal land use as explanatory variables respectively. Considering that the variable characterizing the difficulty of land law enforcement in various provinces and regions (i.e., the administrative land area of each province) in the explanatory variables did not change significantly between 2007 and 2016 and the LDSV test showed that there was no individual fixed effect, a pooled regression was selected for estimation. Table 3 is the estimated results of the number of cases of illegal land use (model 1) and the degree of illegal land use (model 2) affected by the improvement of land law enforcement effectiveness. From the results listed in the table, the related control variables have a significant impact on the dependent variables. Among the explanatory variables, the enhanced deterrence and strength of land law enforcement have played a significant role in reducing the number of illegal land use cases, but failed to better curb the increase in degree of illegal land use; however, with the reduction of the land law enforcement difficulty, the degree of illegal land use has decreased significantly. The following is a detailed analysis of the estimation results of these two models.

**Table 3:** Estimated results of the model.

**Variable**	**Model 1**	**Model 2**
	**Number of illegal land use cases (NUM)**	**Degree of illegal land use (DEG)**
**Constant**	-6155.826	-3.215**
	(4309.485)	(1.596)
**BUR**	-1441.353***	0.274***
	(257.893)	(0.096)
**ADA**	-5.095	0.005***
	(2.929)	(0.001)
**RRC**	-2103.703***	0.834***
	(358.761)	(0.133)
**GPI**	89.433**	0.035**
	(36.826)	(0.014)
**TPO**	0.455***	-5.570e-05***
	(0.042)	(1.550e-05)
**MAR**	-2403.950***	-0.506**
	(657.268)	(0.243)
**N**	310	310
R^2^	0.36	0.30
Adjusted R^2^	0.35	0.29
**F**	28.72	21.65

Note: Standard errors are in parentheses.

*** and ** indicate significant at the 1% and 5% respectively.

### 4.1 The impact of improvement in land law enforcement effectiveness on number of illegal land use cases

The three control variables (GPI, TPO, MAR) in Model 1 all have significant effects on the dependent variables, and are consistent with the existing relevant research results. Specifically, regional economic growth and population increase have a significant positive effect on the increase in the number of illegal cases; and the increase in the level of land marketization will help reduce the occurrence of illegal cases to a certain extent.

From the specific impact of the three explanatory variables (BUR, ADA, RRC) reflecting the improvement of land law enforcement effectiveness on the number of illegal cases, the estimated coefficients of the deterrence and the strength of land law enforcement are all negative, and all pass the significance test of 1%; however, the impact of the difficulty of land law enforcement is not significant enough on the number of illegal cases. It shows that since the implementation of the NLSS, with the improvement of land law enforcement deterrence and land law enforcement strength, the number of cases of illegal land use has significantly decreased. Specifically, compared with the regions without National Land Supervision Bureau stationed, the number of illegal land use cases in the stationed region with strong land law enforcement deterrence will reduce by more than 1,400 correspondingly; for every 1% increase in the registered rate of illegal land use cases, the number of land violation cases will be reduced by more than 2,100. All these indicate that the improvement of land law enforcement effectiveness has played a positive role in curbing illegal land use and reducing the number of illegal cases. In other words, in areas with strong deterrence and great strength of land law enforcement, in order to reduce the risk of being investigated and the cost of illegality, the relevant organizations or individuals will choose to reduce their frequency of illegality, which is consistent with hypothesis 1 and 3.

### 4.2 The impact of improvement in land law enforcement effectiveness on degree of illegal land use

In model 2, three control variables (GPI, TPO, MAR) have significant influence on the dependent variables, and most of them are in line with the existing theoretical judgments. Specifically, economic development and land marketization have obvious promotion and suppressive effects on the degree of illegal land use, which are consistent with the results of model 1. The population size and the degree of illegal land use show a clear negative relationship; this may be because China’s less populated areas are usually underdeveloped or remote areas, where the conflict between land supply and demand in the process of local economic and social development is relatively small. In addition, these areas are often not the focus of supervision, therefore they will show the characteristics of relatively small number of illegal land use cases, but relatively high degree of illegal land use.

From the three explanatory variables (BUR, ADA, RRC) that reflect the improvement of land law enforcement effectiveness, we can see that: First, the estimated coefficient of variable reflecting the deterrence of land law enforcement is significantly positive, indicating that compared with regions without National Land Supervision Bureau stationed, the degree of illegal land use at the stationed region of National Land Supervision Bureau is higher, which verifies hypothesis 4. It means that in the view of potential lawbreakers, although the risk of illegal land use has increased, while controlling the frequency of violations, they will still have a certain fluke mentality, trying to maintain their own interests expanding the profits of each violation, thus increasing the degree of illegal land use. Secondly, the estimated coefficient of variable reflecting the strength of land law enforcement is also significantly positive, indicating that the increase of land law enforcement strength in each region will lead to a significant increase in the degree of illegal land use. Specifically, with each 1% increase in the registered rate of illegal cases, the degree of illegal land use in each region will increase by 0.83 hectares per case, which is also consistent with hypothesis 4. Thirdly, although the estimated coefficient of variable reflecting the difficulty of land law enforcement is significantly positive, the result shows that in areas where the jurisdiction area is relatively small and the law enforcement difficulty is relatively low, the degree of illegal land use is also relatively low. In other words, there is a negative relationship between the reduction of land law enforcement difficulty and the change of illegal land use degree, which is different from hypothesis 4. This may be due to the fact that, compared with the enhancement of law enforcement deterrence and the increase of law enforcement strength, the reduction of law enforcement difficulty is more likely to make the relevant lawbreakers feel the increased risk, thereby prompting them to dare not commit big crimes easily (that is, to increase the degree of illegal land use).

## 5. Conclusions

The phenomenon of illegal land use has always existed in the world, especially in developing countries (e.g. Turkey and Kenya [51, 52]). In order to effectively curb the illegal behaviors, most countries have also made great efforts in improving land law enforcement and governance [53]. While in China, since the reform and opening up, in the process of rapid economic and social development, law enforcement and governance against illegal land use has always been a focus of the Chinese government. Since the enactment of the Land Management Law in the mid-1980s, with the gradual standardization and normalization of land law enforcement, especially the formal implementation of the NLSS, China’s land law enforcement system has become increasingly sophisticated and the effectiveness of law enforcement has been continuously improved. In order to deeply and systematically explore the suppressive effect of improving the effectiveness of land law enforcement on illegal land use in China, this paper expounded the change and improvement characteristics of land law enforcement effectiveness from three aspects: land law enforcement deterrence, land law enforcement difficulty and land law enforcement strength; then, an analytical framework reflecting the impact of the improvement of land law enforcement effectiveness on illegal land use was constructed; furthermore, the mechanism of the enhanced deterrence, the reduced difficulty, and the increase strength of land law enforcement to curb illegal land use were discussed at a theoretical level, and the corresponding research hypotheses were put forward. On this basis, the corresponding empirical tests were carried out using the provincial panel data of Mainland China from 2007 to 2016. The results show that the enhanced deterrence and the increased strength of land law enforcement have played a significant role in reducing the occurrence of illegal land use, the number of illegal cases in the stationed region of National Land Supervision Bureau is significantly less than that in other regions, and the increase in the registered rate of illegal land use is also conducive to the reduction of the number of illegal land use cases; not only that, the enhanced deterrence and the increased strength of land law enforcement will also drive the lawbreakers to "take big risks and commit major cases", leading to an increase in the degree of illegal land use; as the difficulty of land law enforcement decreases, the degree of illegal land use is obviously reduced.

The findings of this study also provide some policy implications for improving relevant systems, effectively curbing illegal activities, and reducing the degree of illegal land use. On the one hand, in the face of the severe illegal land use situation, the Chinese government needs to continuously improve the effectiveness of land law enforcement. The first is to further enhance land law enforcement deterrence through high-frequency, full-coverage inspection and supervision. It is also possible to consider adding local National Land Supervision Bureaus, or exploring the establishment of corresponding agencies or liaison organizations in provinces or regions outside the stationed region of National Land Supervision Bureau. The second is to optimize the allocation of law enforcement resources and formulate differentiated regional law enforcement rules to reduce the difficulty of land law enforcement. Law enforcement zoning can be adopted according to the illegal land use situation in different regions, and reduce the overall difficulty of law enforcement by allocating more resources to areas with serious illegal activities. The third is to constantly innovate the technical means of land law enforcement, establish and improve the multi-party supervision and investigation mechanism of illegal land use, further increase the filing and investigating rate of illegal land use cases to increase land law enforcement strength. On the other hand, it is still necessary to comprehensively deepen reform [54], improve the performance evaluation mechanism of local governments and gradually eliminate the excessive dependence of local development on land economy. At the same time, China can also explore and refine the punishment measures for different illegal land use behaviors, such as distinguishing the level of illegal land use and formulating different punishment measures, to effectively prevent the increase in the degree of illegal land use that may result from the improvement of land law enforcement effectiveness. Moreover, the policy implications above can also bring some enlightenments to the governance of illegal land use in other countries in the world.

Of course, this paper only preliminary discusses the impact of the improvement of land law enforcement effectiveness on illegal land use. Due to the complexity of land law enforcement system and practice, as well as the limitations on the acquisition of relevant data, we only focus on the specific impact of the improvement of land law enforcement effectiveness on illegal land use since the implementation of the NLSS, and the variables selected in this paper to reflect the deterrence, difficulty and strength of land law enforcement are relatively simple. To scientifically reflect the effectiveness of land law enforcement, further relevant theoretical analysis needs to be followed up and more index or data needs to be explored to carry out corresponding empirical researches.

## Supporting information

S1 Data The data used in this article is provided in S1 Data. (XLSX)
